# Effect of Inter-Fragmentary Gap Size on Neovascularization During Bone Healing: A Micro-CT Imaging Study

**DOI:** 10.3389/fbioe.2022.808182

**Published:** 2022-02-23

**Authors:** Zhilun Zhou, Yang Yan, Hao Yu, Guanzhong Yang, Hao Su, Tao Zhang, Yubo Fan, Feng Zhao

**Affiliations:** ^1^ Key Laboratory for Biomechanics and Mechanobiology of Ministry of Education, Beijing Advanced Innovation Center for Biomedical Engineering, School of Biological Science and Medical Engineering, Beihang University, Beijing, China; ^2^ Department of Orthopeadics, Tianjin Hospital, Tianjin, China

**Keywords:** inter-fragmentary gap size, bone healing, neovascularization, vascular perfusion, micro-CT imaging

## Abstract

**Introduction:** Neovascularization of the fracture site is of great importance for bone healing and could be influenced by local mechanical environment such as fixation stability and inter-fragmentary gap size. This study aims to reconstruct the neovascularization of the fracture site and explore the effect of inter-fragmentary gap size on the spatiotemporal structure of vascularity during bone healing.

**Methods:** Osteotomy was performed on 36 Sprague–Dawley (SD) rats on the right tibial diaphysis, and the fracture was given stable fixation with two different inter-fragmentary gap sizes. SD rats received stable fixation with either a small-sized inter-fragmentary gap (FSF1, 1 mm, *n* = 18) or a large-sized one (FSF3, 3 mm, *n* = 18). The left hind limbs were treated as the control group (CON). The animals were killed at different time points (2, 4, and 6 weeks postoperatively, *n* = 6, respectively) for vascular perfusion and micro-CT imaging.

**Results:** (a) At week 2 and 4, FSF1 group showed significantly higher vessel volume ratio (VV/TV) and vessel surface density (VS/TV) values than both CON and FSF3 group; there was no significant difference in either VV/TV or VS/TV values between CON and FSF3 groups. (b) At week 6, both FSF1 and FSF3 groups showed significantly higher VV/TV and VS/TV values than CON group; FSF3 group had a significantly higher VV/TV value than FSF1 group.

**Conclusion:** Different inter-fragmentary gap sizes greatly affect the timing of angiogenesis at the fracture site. Stable fixation with a small inter-fragmentary gap (1 mm) benefits neovascularization at the early stages during bone healing and reconstruction, while stable fixation with a large inter-fragmentary gap (3 mm) delays the occurrence of angiogenesis to a later phase.

## Introduction

Bone is capable of regenerating new osseous tissues at the impaired or defect part (Prendergast and van der Meulen, 2001). Bone fracture healing is a sophisticated physiological process, results of which mainly depend on blood supply (Glowacki, 1998; Carano and Filvaroff, 2003; Tomlinson et al., 2013) and local mechanical environment (Bailón-Plaza and van der Meulen, 2003; Gardner and Mishra, 2003; Epari et al., 2010) of the fracture site.

It has long been recognized that blood supply plays an important role in bone formation since Haller noted in in his book Experimentorum de ossium formatione in 1763: “…the origin of the bone is the artery carrying the blood and in it the mineral elements” (Haller, 1763). Bone fracture is usually accompanied with vessel injury. Bone tissue renewal involves sophisticated mechanisms which are centered on the interaction and coupling between osteogenic and angiogenic events (Hu and Olsen, 2016; Grosso et al., 2017; Peng et al., 2020). Angiogenesis and revascularization at the fracture site are prerequisites for bone healing and come along with the whole healing process, allowing angiogenesis and osteogenesis to be intimately connected and coupled for physiological bone regeneration. In fact, inhibition of angiogenesis can compromise physiological bone healing. It was demonstrated that bone healing was prevented in a murine fracture model receiving angiogenesis inhibitors (Hausman et al., 2001). In another murine osteotomy model, local inhibition of angiogenesis was shown to result in an atrophic non-union (Fassbender et al., 2011).

There are also several mechanical factors that have impacts on bone fracture healing, such as fracture type, fixation stability, and inter-fragmentary gap size (Epari et al., 2010). Mora and Forriol proved that bone repair was affected by both fracture type (transverse or oblique fracture) and fixation stability (rigid or dynamic fixation) *via* testing the increase in bone callus stiffness of an ovine tibial osteotomy model (Mora and Forriol, 2000). Inter-fragmentary gap, as well, has an essential influence on bone repair, as mentioned in Claes et al. (1997) and (2003) experimental and clinical work (Claes et al., 2002). In an ovine metatarsal osteotomy model, an increase in the size of the gap (from 1 to 6 mm) resulted in a significant reduction in the bending stiffness of the healed bones (Claes et al., 1997). In addition, more bone formation and less fibrocartilage tissue were found in sheep with a medium gap sized 2.1 mm than in those with a large one sized 5.7 mm (Claes et al., 2003). Clinically, healing time of fracture increased with an increasing fracture gap size when studying and analyzing 100 cases of tibial shaft fracture (Claes et al., 2002).

In fact, not only does the local mechanical environment have an influence on bone healing but it also affects neovascularization during bone repair. Claes et al. (2003) also found that there was a correlation between gap size and revascularization at the fracture site such that a small gap sized 2.1 mm resulted in more revascularization than that sized 5.7 mm. However, their quantitative analysis of revascularization was mainly limited to a statistical description of two-dimensional (2D) density of the vessels at the fracture site (Claes et al., 2003).

As is known, a major function of blood supply is to transport oxygen and nutrients to different parts of the body. To do this more efficiently, it requires an optimized spatiotemporal vascular network. In addition, it could be deduced that the repair of blood supply is dependent on the reconstruction of an efficient vascular network. Therefore, Zhao et al. (2014) hypothesized that in addition to vessel number, spatiotemporal network formation of neovascularization during bone fracture healing could also be influenced by the mechanical environment. Using vascular perfusion and micro-CT imaging methods (Sinusas, 2004; Savai et al., 2009), Zhao et al. (2014) reconstructed the three-dimensional (3D) structure of the vascular network at the fracture site, testifying that fixation stability, one of the mechanical factors, influenced the timing and 3D structure of neovascularization during bone healing in a murine tibial fracture model. Axial stress/strain (as shown in the group of fracture with stable fixation) would induce more longitudinal neovascularization formation to improve the inter-fragmentary blood fluid connectivity, while shear stress/strain (as shown in the group of fracture with no fixation) would induce more transversal vascular formation (Zhao et al., 2014).

In the current study, another mechanical factor, fracture gap size, was hypothesized to influence the timing and 3D structure of vascularity at the fracture site. Vascular perfusion and micro-CT imaging were utilized to reconstruct the 3D structure of neovascularization at the fracture site and explore the effect of inter-fragmentary gap size on angiogenesis around the fracture during bone healing process (Lu et al., 2006; Zhao et al., 2014; Zhou et al., 2015).

## Materials and Methods

### External Fixator

In this study, a custom-designed circular external fixator, reported previously by Zhao et al. (2014), served as a fixator which could be applied to a fractured Sprague–Dawley (SD) rat’s tibial diaphysis with different inter-fragmentary gap sizes. The fixator mainly consisted of two rings, three connecting sleeves, three shafts, and several nuts and spacers (Zhao et al., 2014). The rings and connecting sleeves were made of aluminum alloy to keep the overall weight of the fixator suitable for SD rats (Zhao et al., 2014). The shafts were made of stainless steel to allow the fixator to maintain adequate strength (Zhao et al., 2014). The overall stiffness of this fixator was 54.11 ± 5.58 N/mm (Zhao et al., 2014).

### Experimental Animals

Thirty-six healthy male SD rats aged 6 weeks with an average weight of 200 g were randomly assigned into two experimental groups. One group (FSF1, fracture with stable fixation and an inter-fragmentary gap of 1 mm) was treated with the newly custom-designed fixator after osteotomy on their right tibial diaphysis of 1 mm, and the other group (FSF3, fracture with stable fixation and an inter-fragmentary gap of 3 mm) received the identical external fixator after osteotomy on the right tibial diaphysis of 3 mm. Both groups were again randomly assigned into three subgroups according to different sacrifice times (2, 4, and 6 weeks postoperatively, *n* = 6, respectively).

All the animals were purchased from the Experimental Animal Center of Beijing University. Animal treatment and care were in accordance with Regulations for the Administration of Affairs Concerning Experimental Animals promulgated by Decree No. 2 of the State Science and Technology Commission of China and the Guiding Principles for the Care and Use of Animals approved by Beijing Government. All protocols were approved by the Animal Care Committee of Beihang University, Beijing, China.

### Surgical Procedure

To perform transverse osteotomy of the tibial diaphysis, each SD rat was weighed before surgery for calculating the dose of 1% sodium pentobarbital (4 ml/kg) for general anesthesia. Under general anesthesia, each SD rat was placed in the supine position. The external fixator was mounted on the SD rat’s right shank *via* Kirschner wires (diameter = 0.8 mm) placed perpendicularly to the longitudinal axis of the tibial diaphysis after preoperative preparation of the skin. Iodophor diluent was applied to disinfect the skin. A 5-mm skin incision was made above the tibial crest, and transverse osteotomy of the tibial diaphysis was then performed. For FSF1 and FSF3, the inter-fragmentary gap was, respectively, set as 1 and 3 mm by using appropriate spacers within the fixator when performing osteotomy. The skin was sutured and covered with a bandage after the procedure.

### Postoperative Care

Postoperatively, the SD rats in both experimental groups were raised individually in cages in the same room. The animals were free to move in the cages. They were given food and water daily. Sawdust was used as bedding material.

### Vascular Perfusion

Each subgroup of the SD rats were killed, and vascular perfusion was performed at 2, 4, and 6 weeks after surgery. At different sacrifice times, the SD rats were anesthetized with 1% sodium pentobarbital (4 ml/kg), and bilateral femoral vessels were exposed after skin incision, which made it convenient to observe and manipulate the process of vascular perfusion. The thoracic cavity of the SD rat was then opened, helping expose the heart. Heparinized saline (100 U/mL) was then injected into the left ventricle until the femoral vessels turned transparent and the liquid flowing out of the right atrium became clear; at the same time, the right atrium was opened to substitute heparinized saline for blood in the entire circulatory system. Afterward, a freshly made barium sulfate suspension (30 g/100 ml) was injected in the same way as heparinized saline into the circulatory system until the femoral vessels turned white and barium sulfate suspension began to flow out of the right atrium.

After vascular perfusion, the hind limbs of both sides were dissected (using a string to ligate above the knee joint to reduce the leakage of barium sulfate suspension inside hind limb vessels) and kept in 4% paraformaldehyde solution at 4°C overnight for fixation. From the next day on, they were placed in 19% tetrasodium EDTA solution at 4°C for 5 days for decalcification (Lu et al., 2006).

### Micro-CT Evaluation

For the quantitative 3D analysis, all the hind limb specimens were scanned in a micro-CT scanner (Skyscan 1076, Belgium). The resolution was set as 9 μm, and scanning was performed at 70 kV and 142 μA, with a 1.0-mm aluminum filter. The images obtained from scanning were then reconstructed using Nrecon software (v.1.6.4.6). The ring artifact correction was set at 8, smoothing was set at 0, and beam hardening correction was set at 30%.

After 3D reconstruction, a circular region of interest, covering the transverse section area of the samples in each image, was set along the tibial diaphysis (4 mm long, taking the osteotomy position as the midpoint) in the micro-CT analyzer software named CTAn (v.2.6) to obtain the values of vessel volume ratio (VV/TV) and vessel surface density (VS/TV).

### Statistical Analyses

For FSF1, the left and right hind limb specimens were named as CON1 group and FSF1 group, respectively. For FSF3, their left and right posterior limb specimens were, respectively, named as CON3 group and FSF3 group. In addition, the left hind limb specimens in both FSF1 and FSF3 (CON1 and CON3) were named as CON group.

Statistical analyses were performed using SPSS software (IBM SPSS Statistics, v.20.0, IBM Corp., New York, United States). An independent-sample *t*-test was used to compare the differences of VV/TV and VS/TV values between CON1 and CON3 groups to ensure the effectiveness of combining both CON1 and CON3 groups as one control group (CON) in the following statistical analysis. Then, one-way ANOVA with LSD post hoc tests was performed to compare the differences of VV/TV and VS/TV values among CON, FSF1, and FSF3 groups. For all the statistical analyses in the study, the level of significance was set at *p* = 0.05.

## Results

### 3D Vascular Reconstruction

**FIGURE 1:**
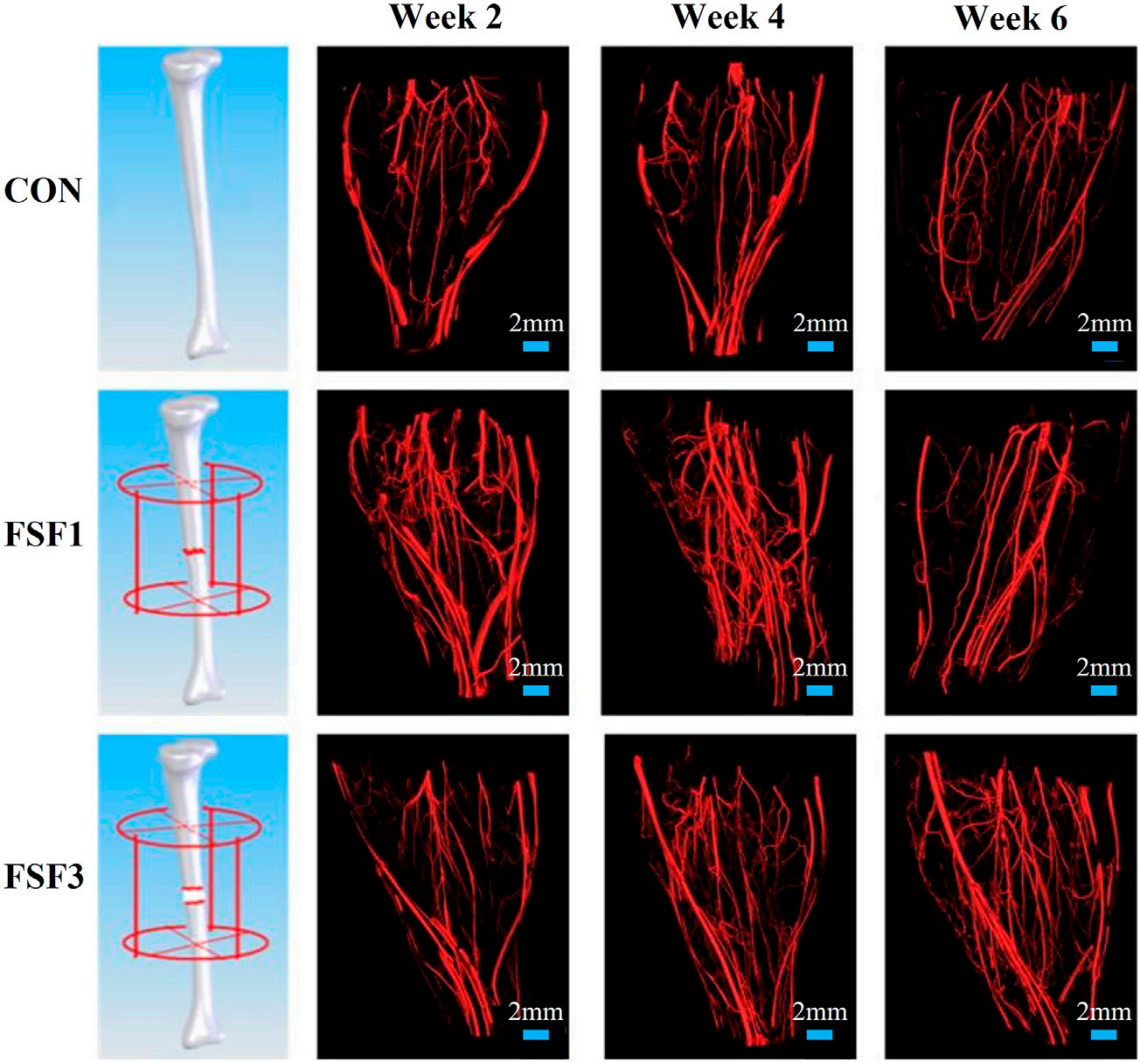
Representative 3D vascular reconstruction images within the soft tissues surrounding the fracture site from micro-CT imaging. At all sacrifice times, the FSF1 group had more vessels, especially microvessels, than the CON group. At week 2 and 4, FSF1 specimens had more vascular distribution than the FSF3 group, and the 3D vascular reconstruction images did not show visible differences between FSF3 and CON groups. At week 6, FSF3 specimens displayed a larger number of microvessels than the FSF1 group.

Representative 3D vascular reconstruction images within the soft tissues surrounding the fracture site from micro-CT imaging are shown in Figure 1. At all sacrifice times, the FSF1 group had more vessels, especially microvessels than the CON group. At weeks 2 and 4, FSF1 specimens had more vascular distribution than the FSF3 group, and the 3D vascular reconstruction images did not show visible difference between FSF3 and CON groups. At week 6, FSF3 specimens displayed a larger number of microvessels than the FSF1 group.

**FIGURE 2:**
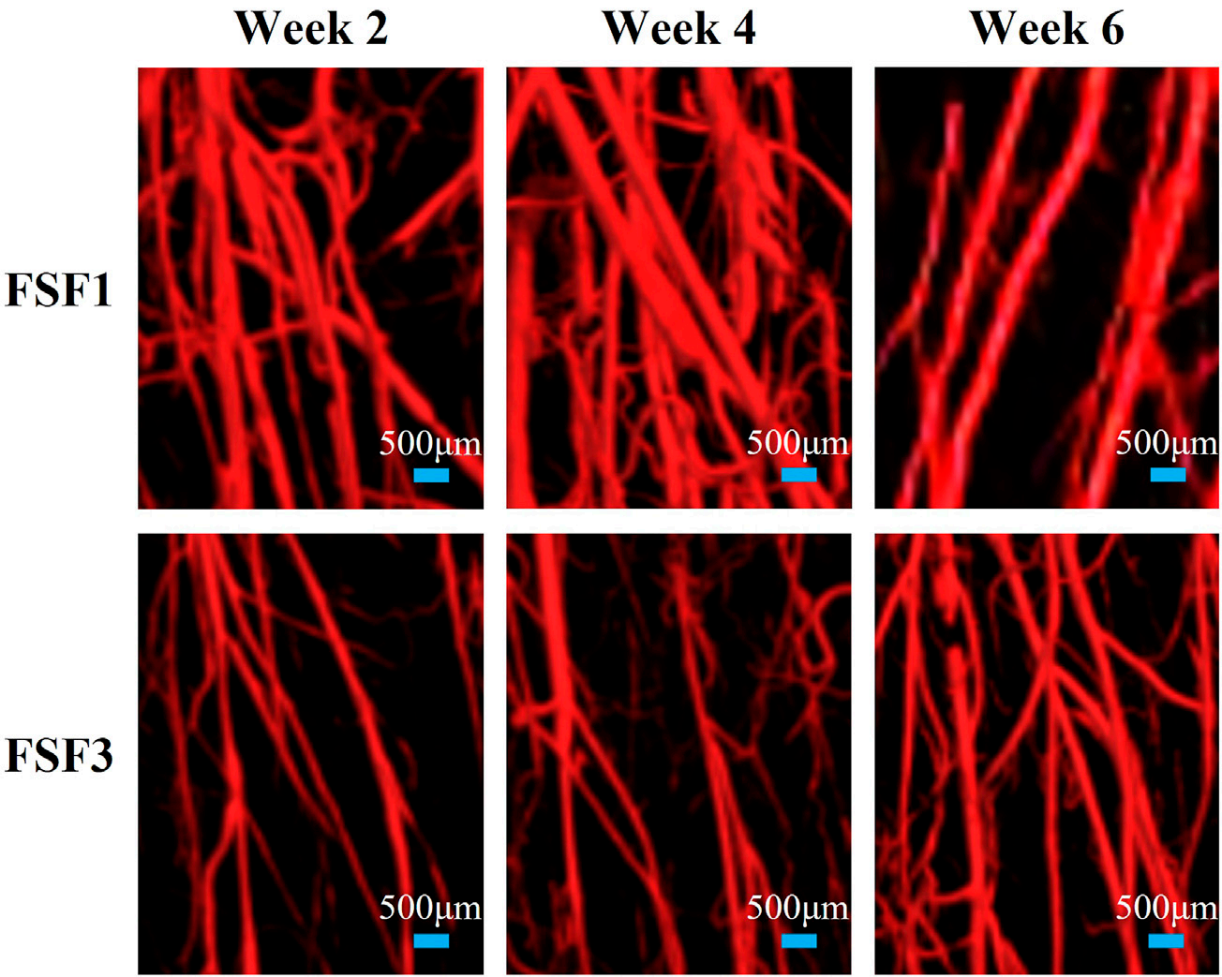
Representative enlarged 3D vascular reconstruction images of FSF1 and FSF3 groups at all sacrifice times.

The 3D images also visually displayed the spatiotemporal structure of neovascularization. Both FSF1 and FSF3 specimens had a similar 3D structure as the CON group, having a much larger number of microvessels in the longitudinal direction along the tibia than in the transversal direction. However, there was a difference in the formation time of the large number of microvessels. In the early phases (week 2 and 4 postoperatively) of the repair process, specimens with 1-mm–sized inter-fragmentary gap had more microvessels than both the control group and the specimens with a 3-mm–sized gap. When the inter-fragmentary gap was sized 3 mm, robust angiogenesis occurred at a later time. There was no distinct difference in the microvessel number between FSF3 and CON groups in the beginning. As the repair time went by, FSF3 specimens had more microvessels in the later phase (week 6 postoperatively) of the healing process. Enlarged 3D vascular reconstruction images (Figure 2) demonstrated the spatiotemporal differences of microvessels between FSF1 and FSF3 specimens.

### Vessel Volume Ratio (VV/TV) and Vessel Surface Density (VS/VT)

**FIGURE 3:**
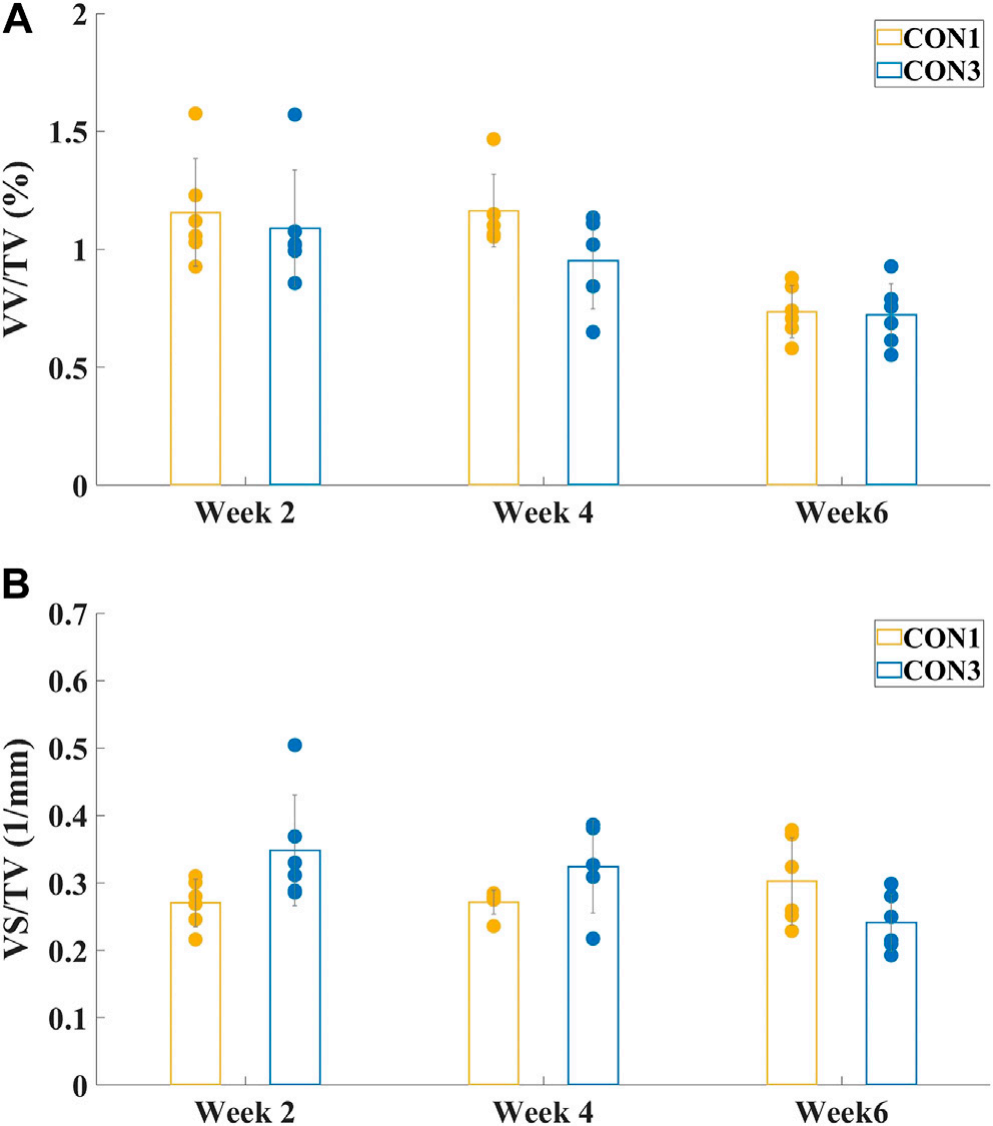
Mean values and standard deviations of vessel volume ratio **(A)** VV/TV and vessel surface density **(B)** VS/TV of both CON1 and CON3 groups at different sacrifice times. There were no significant differences between CON1 and CON3 groups in either VV/TV or VS/TV values at all sacrifice times.

**FIGURE 4:**
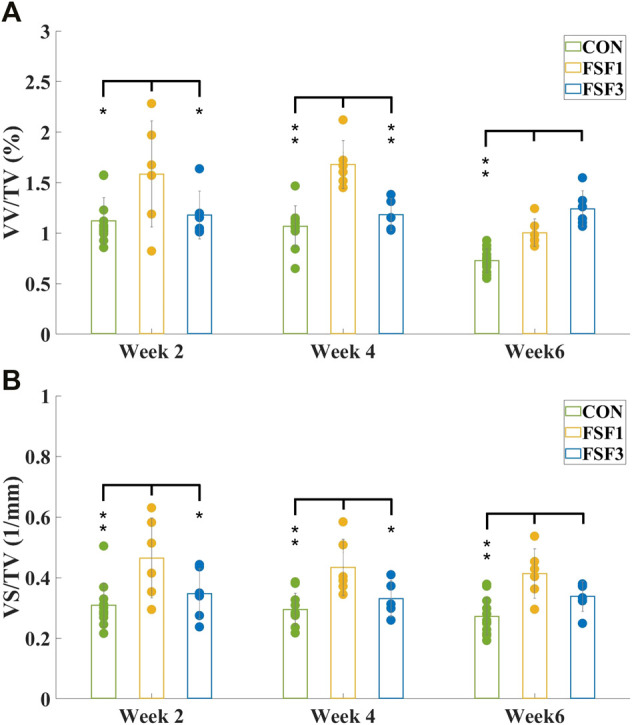
Mean values and standard deviations of vessel volume ratio **(A)** VV/TV and vessel surface density **(B)** VS/TV of CON, FSF1, and FSF3 groups at different sacrifice times. **(A)** VV/TV value of the FSF1 groups was significantly higher than that of the CON group at all sacrifice times; the FSF3 group only displayed a significantly higher VV/TV value than the CON group at week 6, but not at week 2 or 4; the FSF1 group had a significantly higher VV/TV value than the FSF3 group at week 2 and 4, while the FSF3 group had a significantly higher VV/TV value than the FSF1 group at week 6; **(B)** the FSF1 group showed a significantly higher VS/TV value than both CON and FSF3 groups at week 2 and 4; VS/TV value of both FSF1 and FSF3 groups were significantly higher than that of the CON group at week 6. (* indicates *p* < 0.05, ** indicates *p* < 0.01).

VV/TV is the ratio of the vessel volume to the entire volume of the tissue of interest (vessel volume/total volume). VS/TV is the ratio of the vessel surface to the entire volume of the tissue of interest (vessel surface/total volume). The mean values and standard deviations of both VV/TV and VS/TV values for all experimental group samples obtained by micro-CT analyzer CTAn software (v.2.6) are presented in Figure 3 and Figure 4.

Figure 3 showed the mean values and standard deviations of VV/TV and VS/TV values of both CON1 and CON3 groups at different sacrifice times. There was no significant difference between CON1 and CON3 specimens in either VV/TV or VS/TV value (*p* > 0.05) at all sacrifice times. Thus, CON1 and CON3 groups could be combined as one group, CON, for the following one-way ANOVA.

Figure 4 showed the mean values and standard deviations of VV/TV and VS/TV values of CON, FSF1, and FSF3 specimens at different sacrifice times. At week 2, the FSF1 group had a significantly higher VV/TV value than both FSF3 (*p* < 0.05) and CON groups (*p* < 0.05); and there was no significant difference in VV/TV value between FSF3 and CON groups (*p* > 0.05). At week 4, VV/TV value of the FSF1 group was significantly higher than that of both FSF3 (*p* < 0.01) and CON groups (*p* < 0.01); and there was no significant difference in VV/TV value between FSF3 and CON groups (*p* > 0.05). At week 6, the FSF3 group had a significantly higher VV/TV value than both FSF1 (*p* < 0.01) and CON groups (*p* < 0.01); and the FSF1 group had a significantly higher VV/TV value than the CON group (*p* < 0.01).

At week 2, VS/TV value of the FSF1 group was significantly higher than that of both FSF3 (*p* < 0.05) and CON groups (*p* < 0.01); and there was no significant difference in VS/TV value between FSF3 and CON groups (*p* > 0.05). At week 4, the FSF1 group had a significantly higher VS/TV value than both FSF3 (*p* < 0.05) and CON groups (*p* < 0.01); and there was no significant difference in VS/TV value between FSF3 and CON groups (*p* > 0.05). At week 6, both FSF1 and FSF3 groups had a significantly higher VS/TV value than the CON group (*p* < 0.01); and there was no significant difference in VS/TV value between FSF3 and FSF1 groups (*p* > 0.05).

## Discussion

The 3D reconstructed micro-CT images and the numerical values obtained from the images gave us insights into the number, structure, and timing of neovascularization at the fracture site during the bone healing process. The effect of inter-fragmentary gap size on neovascularization could be investigated *via* comparing the vascular network at the fracture site between FSF1 and FSF3 specimens in this study.

From the numerical values such as vessel volume ratio (VV/TV) and vessel surface density (VS/TV) calculated from the micro-CT images, we found that the FSF1 group had more vessels visually and significantly higher VV/TV and VS/TV values than the CON group at all sacrifice times; and at weeks 2 and 4, the FSF1 group had more vessels visually and a significantly higher VV/TV value than the FSF3 group (Week 2: VV/TV_FSF1_ = 1.59 ± 0.53% > VV/TV_FSF3_ = 1.18 ± 0.24%, *p* < 0.05; Week 4: VV/TV_FSF1_ = 1.68 ± 0.24% > VV/TV_FSF3_ = 1.18 ± 0.16%, *p* < 0.01). Both findings indicated that during the early phases (week 2 and 4) of bone repair, a small-sized inter-fragmentary gap (1 mm) could elicit more neovascularization than a large gap (3 mm). At the same time, the FSF1 group had a significantly higher VS/TV value than the FSF3 group at 2 and 4 weeks postoperatively (Week 2: VS/TV_FSF1_ = 0.46 ± 0.13 mm^−1^ > VV/TV_FSF3_ = 0.35 ± 0.08 mm^−1^, *p* < 0.05; Week 4: VS/TV_FSF1_ = 0.43 ± 0.09 mm^−1^ > VV/TV_FSF3_ = 0.33 ± 0.06 mm^−1^, *p* < 0.05), also suggesting that a small inter-fragmentary gap (I mm) was more beneficial to angiogenesis than a large gap (3 mm) since newly grown microvessels would contribute to a larger vessel surface. All of the above mentioned results agreed with what Claes and his coworkers held that a small gap (2.1 mm) resulted in more revascularization at the fracture site than a large one (5.7 mm) (Claes et al., 2003). At week 6 postoperatively, the FSF3 group showed more vessels visually and had a significantly higher VV/TV than the FSF1 group (VV/TV_FSF3_ = 1.33 ± 0.19% > VV/TV_FSF1_ = 1.00 ± 0.13%, *p* < 0.01), inferring that angiogenesis started being robust with a large gap of 3 mm only in a later phase (week 6) of bone healing.

From the 3D reconstructed micro-CT images, we found that both FSF1 and FSF3 groups shared a similar vascular structure with the CON group, with a larger number of newly grown microvessels (at early or late stages of bone repair, respectively) in the longitudinal direction of tibia rather than in the transversal direction, suggesting that stable fixation could avoid shear movement between the fracture ends. This was consistent with our and others’ previous findings when investigating the effect of fixation on angiogenesis at the fracture site during bone healing (McKibbin, 1978; Wu et al., 1984; Zhao et al., 2014; Zhou et al., 2015). Furthermore, it could be deduced that fixation type/stability affects the 3D structure of the vascular network at the fracture site, and changing inter-fragmentary gap sizes tends to have little influence on the 3D structure of the vessels.

Although different inter-fragmentary gap sizes under stable fixation might only have limited effect on the 3D structure of neovascularization at the fracture site, the results of this study showed that the inter-fragmentary gap size would play an essential role in the timing of neovascularization. The FSF1 group had more vessels, especially microvessels, than the CON group at all sacrifice times. At week 2 and 4, the FSF1 group had more vascular distribution than the FSF3 group, and the 3D vascular reconstruction images did not show visible differences between FSF3 and CON groups, whereas at week 6, the FSF3 group showed an even larger number of microvessels than the FSF1 group. This visual difference in timing of angiogenesis between the FSF1 and FSF3 groups could be the results of the different time periods needed to reconstruct the inter-fragmentary blood fluid connectivity due to different gap sizes. Compared to a 3-mm inter-fragmentary gap size, it took less time for the remaining vessels at the fracture ends with a 1-mm gap size to “get together” and elicit angiogenesis at an earlier phase (week 2 and 4) during bone healing. It could be deduced that it might take longer for the existing vessels from the fracture ends with a 3-mm–sized gap to reconnect, thus delaying the angiogenesis and resulting in neovascularization of the FSF3 group only being relatively more robust at a later phase of bone healing.

The choice of the two inter-fragmentary gap sizes (1 and 3 mm) in this study was based on the size of SD rats’ tibia and a previous work in our laboratory (Zhao et al., 2014), from which we have shown that stable fixation with an inter-fragmentary gap of 1 mm could promote longitudinal vascular formation. Adding a 3-mm–sized inter-fragmentary gap in this study provided us with information that a larger gap would delay neovascularization during bone healing. It would also be interesting in future studies to see if a gap that is smaller than 1 mm or even no inter-fragmentary gap size would benefit the vasculature at the fracture site since smaller gap size could result in fewer micromovements between the fracture ends, which might promote the inter-fragmentary blood fluid reconnection, especially during early phases of recovery.

This study mainly focused on how different biomechanical environments created by an external fixator with different inter-fragmentary gap sizes affect neovascularization during bone healing. Quantitative analysis of the effect of different structures from different gap sizes on local mechanical distribution will be needed in the future. Our laboratory is currently working on the finite element simulation of fractured bone with the external fixator along with different inter-fragmentary gap sizes. Ultimately, it is challenging to differentiate and separate structural and mechanical contributions of the fixation to the neovascularization since they are closely interconnected. Fixation with different inter-fragmentary gap sizes would influence the local dynamics at the fracture site, which would then have an impact on the reconstruction of the blood flow network.

Vascular perfusion and micro-CT imaging made it possible for researchers to observe the vascular distribution and obtain key parameters (VV/TV and VS/TV) of the vascular network (Sinusas, 2004; Jeswani and Padhani, 2005; Savai et al., 2009). The high resolution solved the problems of both visualization and complicated calculation of the entire circulatory system, especially the microvessels. Although large animal models (e.g., canine or ovine) resemble humans more in lower extremity loading patterns especially during standing and walking, it would be less feasible to perform whole body vascular perfusion on a large animal. In addition, standard CT or MRI used for imaging large animal models usually does not have the high resolution to allow imaging of microvessels at the fracture site. In order to have better image quality of the vasculature, the hind limb specimens were decalcified in tetrasodium EDTA solution at 4°C for a total of 5 days, which made it challenging to reconstruct bone images and evaluate bone formation at the fracture site. Another limitation with the techniques used in this study could be that it is hard to distinguish newly grown vessels from the existing ones in micro-CT images. Because of this, immunohistochemical methods (Lu et al., 2011), which would help identify the neovascularization area within the region of interest, could be used together with micro-CT imaging in future studies.

## Conclusion

3D reconstructed micro-CT images and quantitative evaluation of the vascular parameters demonstrated that neovascularization at the fracture site and its spatiotemporal structure are apparently influenced by different inter-fragmentary gap sizes of bone fracture. Under stable fixation, different gap sizes would have great impact on the timing of angiogenesis, while spatial structure of vascularity at the fracture site would be less affected. Stable fixation with a small gap sized 1 mm benefited neovascularization at early phases (week 2 and 4) during bone repair, while stable fixation with a large gap sized 3 mm delayed the occurrence of angiogenesis to a later phase (week 6) of bone healing.

## Data Availability

The raw data supporting the conclusions of this article will be made available by the authors, without undue reservation.
